# Improvement of Ball Mill Performance in Recycled Ultrafine Graphite Waste Production for Carbon Block Applications

**DOI:** 10.1021/acsomega.3c02688

**Published:** 2023-07-21

**Authors:** Waroot Kanlayakan, Siraprapa Lhosupasirirat, Chonradee Amnatsin, Nattarut Verojpipath, Boonsueb Pragobjinda, Toemsak Srikhirin

**Affiliations:** † School of Materials Science and Innovation, Faculty of Science, Mahidol University, Nakhon Pathom 73170, Thailand; ‡ Department of Physics, Faculty of Science, Mahidol University, 272 Rama VI Road, Ratchathewi District, Bangkok 10400, Thailand; § Research Network of NANOTEC at Mahidol University, National Nanotechnology Center, Pathum Thani 10400, Thailand; ∥ Thai Carbon & Graphite Co., Ltd., 15/2 Phutthamonthon Sai IV Road, Kratumban District, Samutsakorn 74130, Thailand

## Abstract

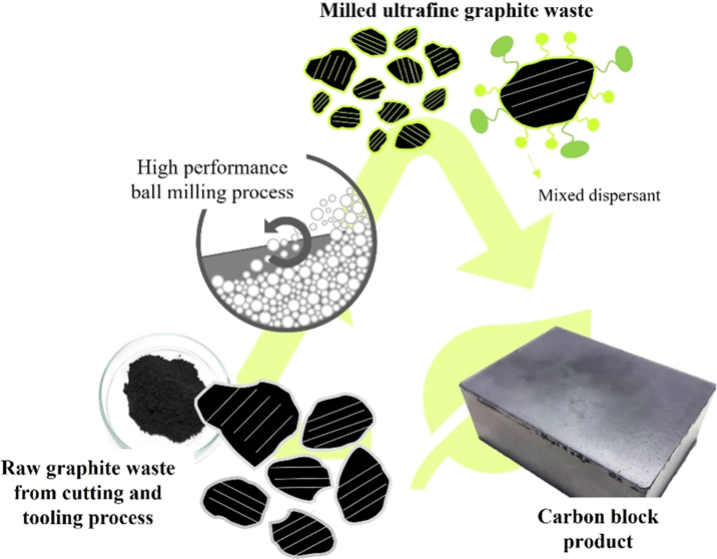

A carbon block is a carbonaceous material used in various applications such as bearings, mechanical seals, and electrical brushes. This work aims to fabricate carbon blocks from industrial graphite waste, a residue from the cutting and tooling process of graphite block production. The ball milling process was used to fabricate ultrafine graphite waste to enhance the packing of carbon blocks. The milling performance was profoundly affected by dispersing agents in which sodium dodecyl sulfate (SDS), lignosulfonate (LS), and mixed dispersant (LS–SDS) were applied. The results showed that LS–SDS had the best milling performance, the greatest grinding index, and a flowable slurry, indicating the potentiality of this formulation for the environmentally friendly manufacture of ultrafine graphite waste. Carbon blocks were prepared from oven-dried ultrafine graphite waste, which was mixed with amorphous carbon and pitch. This carbon mixture was formed a block by compaction before carbonization and impregnation. The density of the fabricated carbon blocks increased from 1.76 to 1.83 g/cm^3^ after impregnation along with the increase in hardness, flexural strength, and reduction in electrical resistivity from 83, 62 MPa, and 40 μΩ m to 88, 81 MPa, and 39 μΩ m, respectively. The physical properties of carbon blocks prepared from ultrafine graphite waste were comparable to the properties of typical pristine carbon products.

## 1 Introduction

Graphite is a unique material due to its electrical and thermal conductivity, chemical inertness, and lubricant properties. The structure of graphite consists of the multilayer stacking of carbon atom planes with conjugated pi-bond systems. It can be categorized as natural graphite, produced from mining, and synthetic graphite, produced from the graphitization of carbon precursors.^1^ A graphite block is a synthetic graphite, manufactured from a mixture of carbon fillers such as coke, mesocarbon microbeads (MCMBs), and natural graphite with a pitch binder by hot mixing, pulverizing, compaction, carbonization, and graphitization before the graphite blocks are machined to a precise shape.^2^ These cutting and tooling processes generate a large quantity of graphite waste, also known as “graphite scrap” or “secondary synthetic graphite,” which is currently estimated to be 14 600 MT annually.^3^ Nowadays, the usage of graphite scrap as a recycling material is required for sustainable manufacturing and production cost-saving for material and waste disposal costs. For example, graphite scrap has been used as electrodes for Li-ion batteries, electrically conductive particles in bipolar plates, fillers for increasing thermal and physical properties in phase change materials (PCMs), carbon precursors to fabricate isotropic graphite blocks, etc.^3,4^

A carbonaceous material known as a carbon block has long been used for electrical brushes, bearings, and face seal components. Similar to graphite blocks, carbon blocks are produced using a heat treatment process that finishes with carbonization. The properties of carbon blocks depend on the composition, type, and size of the carbon filler and the temperature of carbonization.^5,6^ Carbon blocks can be categorized, following the ASTM D8075-16 standard, as coarse grain, medium grain, fine grain, superfine grain, and ultrafine grain. These grain sizes are directly controlled by the size of the carbon filler.^7^ The preparation of a block with a smaller grain size yields a product with higher density, hardness, and flexural strength, especially for ultrafine products with grain sizes less than 10 μm. As a result, the production of ultrafine graphite scrap with a controlled particle size plays a crucial role in the fabrication of carbon blocks.^6,8,9^

Raw graphite scrap from cutting and tooling processes normally has a high degree of purity and crystallinity due to the high graphitization temperature of graphite blocks and has larger particle sizes, ranging from 1 to more than 100 μm, which is unsuitable for the production of ultrafine grain products. The ball milling process is a particle size reduction method, utilizing impaction and shearing of ceramic balls inside the milling chamber to break particles to a smaller size with a high output capacity, which is often applied to manufacture ultrafine graphite particles. However, improper control of the milling process can cause viscosity build-up, which prolongs the process and reduces milling efficiency. The increase of viscosity is a result of an increased surface area of the particle during the milling period. The higher surface area raises the van der Waals interaction between particles and promotes a network of material aggregation.^10^ Generally, chemical additives such as dispersants have been used to decrease the viscosity of the slurry and improve milling performance. The dispersing agents can be absorbed on the particle surface and act as stabilizers through electrostatic and steric stabilization, which decrease slurry viscosity and improve milling efficiency.^11^

Typically, researchers have mostly concentrated on laboratory-scale ball milling techniques for producing ultrafine particles, however the process may change after industrial upscaling. In this work, ultrafine graphite scrap is produced using pilot-scale ball milling in an actual industrial production line. In order to find suitable conditions for the production of ultrafine graphite scrap with high solid loading and low viscosity, different types of dispersants were screened to select the most suitable additive by monitoring the particle size reduction, slurry appearance, and grinding index. Milled ultrafine graphite waste was used as a carbon filler to fabricate carbon block products through product-forming processes. The carbon blocks were also enhanced by the impregnation process to produce a better-quality product. The microstructures and the properties of carbon block products before and after impregnation were characterized. Finally, the effects of dispersant formulations on the characteristics of ultrafine graphite scrap and the properties of carbon blocks were also investigated in order to evaluate the potential for using dispersants in the real manufacturing process.

## 2 Experimental Section

### 2.1 Materials and Chemicals

Raw graphite scrap (GS), passing sieve #60 (<250 μm), from the cutting process of various grade isotropic synthetic graphite blocks, lignosulfonate (LS), and amorphous carbon (*d*_50_ = 4–5 μm) was provided by Thai Carbon & Graphite Co., Ltd. (TCG). Dispersants from common surfactants, anionic sodium dodecyl sulfate (SDS, ≥98%, GPR RECTAPUR), cationic cetyl trimethyl ammonium bromide (CTAB, ≥99%, Sigma-Aldrich), nonionic Triton X-100 (solution, OmniPur), and polymeric sodium polyacrylate (PAA, average *M*_w_ ∼ 2100, Sigma-Aldrich) were used as received. Solvents such as isopropyl alcohol (IPA), ethanol (EtOH), and acetone were also purchased from RCL Labscan Limited.

### 2.2 Dispersant Screening

43 wt % graphite scrap slurries with different dispersant types (anionic (LS and SDS), cationic (CTAB), nonionic (Triton X-100), and polymeric (PAA)) were prepared from 120 g of graphite scrap powder and 160 mL of water with 1.2 g of the dispersant (1 wt % of graphite scrap weight). The dispersion, sedimentation, and viscosity of graphite scrap slurries were used as criteria to select the suitable dispersant. The slurries were mixed by several stirrings before the viscosity measurement by a DVE Brookfield viscometer three times with the following condition: spindle #62 and fixed rotational speed at 60 rpm and the dispersion or sedimentation test by setting aside for 24 h.

### 2.3 Ball Milling Process

A pilot-scale ball mill with a 25 L ceramic chamber, containing 50 kg of four mixed-size ZrO_2_ balls (10, 15, 20, and 25 mm with a ratio of 1:1:1:1 by wt), was run with a fixed rotation speed of 40 rpm. First, 30 g of the dispersant (1 wt % of graphite powder) was completely dissolved in 4 kg of water before adding 3 kg of raw graphite scrap powder, determining a solid loading of 43 wt % approximately. All components were stirred in a container before putting in the milling chamber and running the process. Dispersant types were varied to single dispersant (SDS and LS) and mixed dispersant (LS–SDS) systems with a ratio of 1:1 by wt. (15 g of LS and 15 g of SDS). The milled graphite scrap slurries were sampled at different milling times (1, 2, 4, 6, and 24 h) to observe the reduction of particle size. After 24 h, the slurries were removed and dried at 120 °C for 2 days before pulverizing with a pin mill to keep them in the powder form.

### 2.4 Carbon Block Product Preparation

The carbon blocks were prepared from 30 wt % amorphous carbon, 29 wt % ultrafine graphite scrap, and 41 wt % coal tar pitch. All compositions were blended with a hot mixer at 180 °C. The carbon mixture was cooled down at room temperature and pulverized with a pin mill to form the carbon aggregate before drying at 60 °C for 2 days to remove the humidity. 550 g of dried mixture powder was pressed with die compaction at a pressure of 1000 psi to obtain a green body block with dimensions of 40 cm × 80 cm × 100 cm. The block was carbonized at 950 °C, followed by the impregnation process. Finally, the product properties in terms of the density, hardness, flexural strength, and electrical resistivity of fabricated carbon block products were measured.

### 2.5 Characterization and Measurement

#### 2.5.1 Particle Size Analysis and Grinding Index

The particle size of the graphite scrap was characterized by a laser diffraction technique (Malvern Mastersizer Micro) and reported in terms of volume-based size parameters (*d*_10_, *d*_50_, and *d*_90_). The measurements were performed three times on each sample of milled slurries that were collected from three distinct locations inside the mill chamber.

The grinding index, a simple criterion for quantified industrial milling efficiency based on particle breakage, was used to determine milling efficiency.^12^ It was calculated from %passing a specified size of the raw particle and the milled product following the equation below.
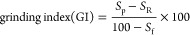
1where *S*_R_ and *S*_P_ were %passing a specified size of the raw and milled particles, respectively. In this work, 10 μm was chosen as the required size due to an ultrafine size. The grinding index was reported between 0 and 100%, which can be interpreted as no size reduction when GI = 0%, and the particle size of the milled particles was completely below 10 μm when GI = 100%.

#### 2.5.2 Crystalline Analysis

The crystallinity of the graphite scrap was identified by X-ray diffraction (D8 discover, Bruker, Germany) using CuKa radiation with λ = 1.5406. The running condition was followed by step size of 0.01° and 2θ range of 10–90°. The interlayer spacing (*d*_002_) was determined from 2θ at 26°, which reflected the 002 plane by Bragg’s law (eq 2). The crystalline size (La) and stacking height (Lc) were identified from the full width at half-maximum (FWHM) of 110 and 002 reflections by the Scherrer formula (eqs 3 and 4, respectively). The graphitization degree was evaluated by the Franklin equation (eq 5).^13,14^
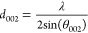
2
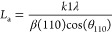
3
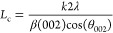
4
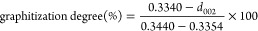
5where *k* is a constant with *k*1 = 1.84 and *k*2 = 0.94 and β is the full width at half-maximum (FWHM) of the diffraction peak.

#### 2.5.3 Scanning Electron Microscope (SEM)

The morphology of graphite scrap particles and carbon block products was characterized using a scanning electron microscope (SEM, JSM-IT500) and a field emission scanning electron microscope (FESEM, JSM-7610FPlus) at an accelerating voltage of 10 kV. For graphite scrap particles, a 0.05 wt % graphite suspension with 0.025 g of graphite powder and 50 mL of isopropyl alcohol (IPA) was prepared. 20 μL of the suspension was dropped on a silicon wafer and suddenly heated in an oven at 110 °C for 2 days. For carbon block products, a cubic sample of 1 cm × 1 cm × 1 cm was prepared by cutting the blocks. The perpendicular surface to the compression direction was polished with wet sandpaper 1200 mesh. Then, all of the samples were dried at 110 °C for 2 days to eliminate the humidity before the observation.

#### 2.5.4 Surface Properties of Graphite Scrap Particles

The surface property of graphite scrap powder was determined by contact angle measurement and the redispersion method. A cylindrical tablet of graphite powder with a diameter of 2 cm was prepared for the contact angle technique. 2 μL of water was dropped on the sample three measurement times to obtain the average contact angle. For the redispersion method, 1 g of the powder was redispersed in 5 mL of water by the sonication method.

#### 2.5.5 Product Properties

The density of the carbon block product was calculated based on the physical measurement of the weight and the dimensional volume after the product-forming process. The carbon block products were cut into six small specimens with dimensions of 1 cm × 1 cm × 10 cm before testing of product properties. The SATO hardness tester model *d* is used to determine the shore hardness. Electrical resistivity was obtained using a four-point test device. Flexural strength was measured by a three-point loading method by a Lloyd Instruments/Ametek EZ50 universal testing machine. The average of all product properties was evaluated from the six specimens.

## 3 Results and Discussion

### 3.1 Characteristics of Raw Graphite Scrap

Raw graphite scrap is a waste material generated during the cutting and tooling process of isotropic synthetic graphite blocks on the production line of Thai Carbon and Graphite Co., Ltd. The basic particle characteristics such as particle size and distribution, morphology, crystallinity, and surface properties were characterized before the manufacturing process to identify the initial qualities of the received graphite waste.

**Figure 1:**
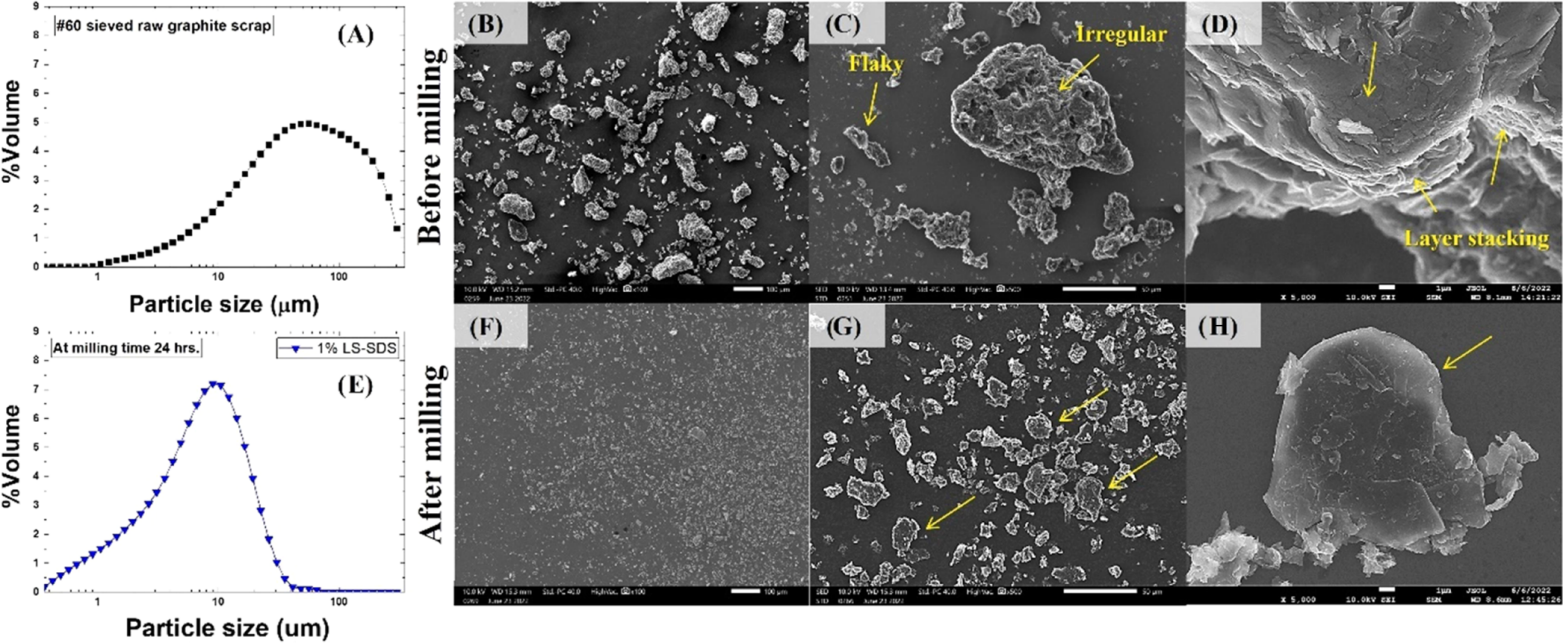
Particle size distribution of raw graphite scrap, passing sieve #60 (A) and ultrafine graphite scrap after milling for 24 h with a mixed dispersant (LS–SDS) (E). The morphology of raw graphite scrap (B–D) and milled ultrafine graphite scrap (F–H) at different magnifications.

Figure 1A illustrates the particle size distribution of raw graphite scrap after passing sieve #60 using the laser diffraction method (Malvern Mastersizer Micro). The result displayed a volume-based particle size distribution, which showed a broad distribution with *d*_10_, *d*_50_, and *d*_90_ values of 7, 39, and 138 μm, respectively. The particle size and distribution also corresponded to the low-magnification (100×) SEM image in Figure 1B. The particles could be large to more than 100 μm and small to less than 10 μm, while the majority of the particle population had a particle size between 30 and 50 μm. The higher-magnification SEM image (Figure 1C) revealed the morphology of the graphite scrap, which was an irregular shape in the large particles and a flaky shape in the small particles, as pointed by arrows. Multilayer stacking of graphene, a general characteristic of graphite, was also noticed in raw graphite waste, as seen following the arrow in a high-magnification (5000×) FESEM image (Figure 1D). The results demonstrated the aggregates from the small-sized graphene layers, stacked on the surface and internal structure of graphite waste.

**Figure 2:**
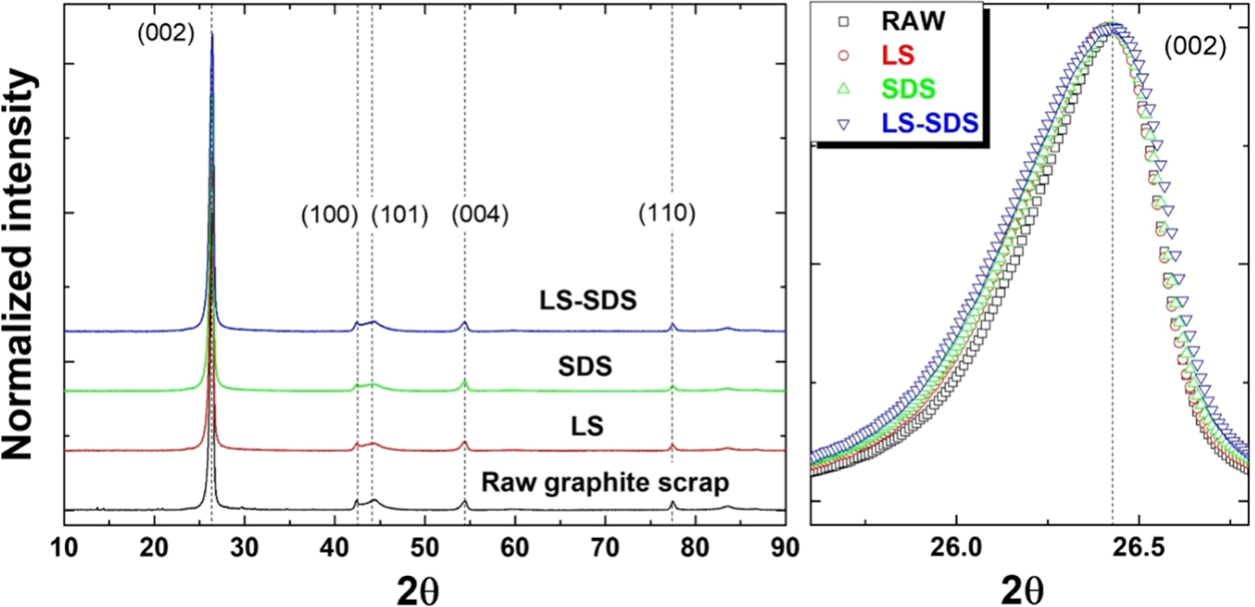
XRD pattern and (002) reflection of graphite scrap before and after the milling process with different dispersant formulations.

Figure 2 shows the crystallinity of the raw graphite scrap as determined by the X-ray diffraction technique (XRD). The peaks were detected at 2θ° of 26, 42, 44, 54, and 77°, referring to (002), (100), (101), (004), and (110) reflections, respectively. This pattern confirmed the reflection of the graphitic structure of the raw graphite scrap.^14^Table 1 lists the crystalline characteristics, including internal spacing (*d*_002_), crystalline size (*L*_a_), stacking height (*L*_c_), and degree of graphitization of the graphite scrap. The *d*-spacing of raw graphite waste was calculated using Bragg’s equation to be roughly 3.37 nm. The graphitization degree, estimated using the Franklin equation from the interlayer spacing, was found to be around 82%. This demonstrated the existence of structural disorder in raw graphite scrap due to the graphitization temperature during the graphite block production. An extreme temperature can produce a high graphitic structure but lower the physical properties of graphite block, which is not suitable for commercial use.^3^ Crystalline sizes of raw graphite scrap particles were calculated from Scherrer’s equation. The results showed that the lateral crystalline size (*L*_a_), which was determined from the (110) reflection, was 416 nm, and the stacking height (*L*_c_), which was identified from the (002) diffraction peak, was 192 nm. These crystallite sizes were significantly less than the particle size, suggesting an aggregation of stacked graphene in different orientations, which corresponded to the FESEM result.^15^

**Table 1:** Crystalline Parameters Such as *d*-Spacing, %Graphitization, the Crystallite Size of the Basal Plane (*L*_a_), and Stacking Height (*L*_c_) of Graphite Scrap Particles before and after the Milling Process for 24 h with Different Dispersant Formulations

crystalline parameters	*d*_002_ (Å)	%G	*L*_c_ (nm)	*L*_a_ (nm)
raw graphite scrap	3.37	82	192	408
LS	3.37	81	179	386
SDS	3.37	81	172	379
LS–SDS	3.37	81	155	365

Generally, the water contact angle measurements of particles were frequently used to identify the surface properties of the particles.^16^ The particle is compacted to a tablet before the test. However, due to the rapid penetration of water drops into the surface of the raw graphite tablets, this approach was not suitable for raw graphite waste. As a result, the dispersion method, which involved suspending graphite particles in liquids with various polarities, was applied in place of contact angle measurement. The stability of graphite particles, dispersed in the solvent, allowed for the observation of surface properties. The stable dispersion was created in a good solvent with surface energies comparable to those of raw graphite scrap. In contrast, sedimentation occurred in an unfavorable solvent where the polarities of the particles and the solvent were incompatible. The stability results of graphite scrap dispersion along with the time in the solvents with different polarities as indicated by the polarity index (PI) are presented in Table 2. Low-polarity solvents like EtOH (PI = 4.3^17^) and IPA (PI = 3.9^17^) provided stable graphite scrap dispersions, while high-polarity solvents like water (PI = 10.2^17^) and acetone (PI = 5.1^17^) produced unstable suspensions and sedimentation occurred clearly after leaving for only 5 min. Although the majority of the graphite particles were precipitated in polar solvents, some dispersed graphite particles were still observable due to the low gravitational forces of the small particles in the particle population. Therefore, because of their rapid sedimentation and unstable suspension, forming in polar solvents, it was indicated that raw graphite scrap particles exhibited hydrophobic nature, which was similar to pristine graphite.^18^

**Table 2:**
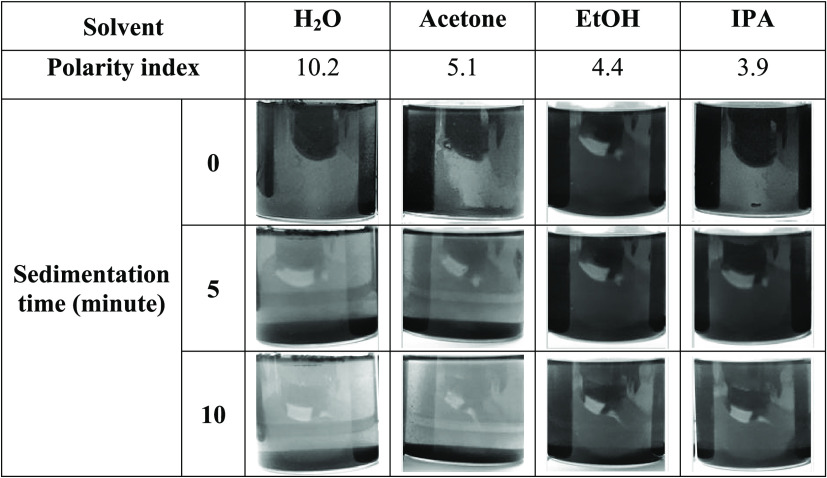
Dispersibility Tests of Raw Graphite Scrap in Solvents with Different Polarity Indices

The investigation revealed the micron-sized raw graphite scrap with an initial particle size, *d*_50_, of 39 μm, a broad distribution, a highly crystalline structure, and a hydrophobic surface, which easily created an aggregation, resulting in a highly viscous slurry. It is well known that the use of dispersants could solve this problem. Therefore, suitable additives were basically screened from different types of dispersants before applying them in the milling process.

### 3.2 Dispersant Screening

Herein, the dispersants are used as surface stabilizers for graphite scrap waste particles to prevent aggregation upon milling and reduce the slurry viscosity. The alignment and distribution of dispersant molecules on the surface of graphite particles are influenced by the dispersant kinds, functional groups, molecule size, and particle surface property. As a result, finding the right dispersant formulation was crucial for ultrafine graphite scrap production by ball milling. Typical dispersants for the carbon base material such as SDS (anionic), LS (anionic), CTAB (cationic), Triton X-100 (nonionic), and PAA (polymeric) were screened. The dispersibility (creating a stable suspension) and low-viscosity slurry (producing a flowable slurry) were used as indicators for this dispersant selection, as shown in Table 3. A suitable dispersant should offer a strong stabilization effect, preventing the formation of aggregates, a low level of sedimentation formation, and flowable slurries with low viscosities.

**Table 3:**
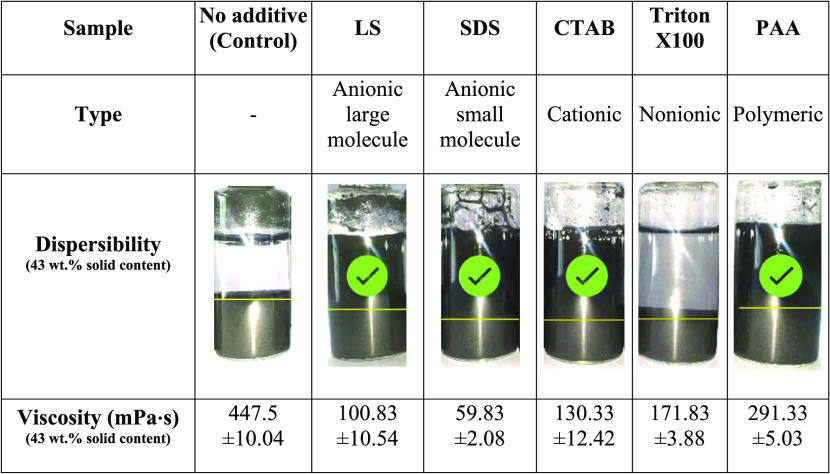
Presented the Dispersibility Test and Viscosity Characteristics of Graphite Scrap Slurry with 43 wt % Solid Loading and Different Dispersant Types: Anionic (LS and SDS), Cationic (CTAB), Nonionic (Triton X-100), and Polymeric (PAA)

Graphite scrap slurries with 43 wt % solid loading were prepared from raw graphite scrap particles, water, and 1 wt % dispersant (percent of powder weight) before the sedimentation test and viscosity measurement. The results revealed significant sedimentation of nonstabilized aggregates in the suspension without additives. The solid and aqueous liquid segments were clearly separated after leaving for a while, indicating an aggregation of graphite particles due to a high van der Waals interaction between hydrophobic surfaces. The addition of dispersing agents can produce electrostatic and steric stabilization against the van der Waals interaction, which increased the slurry suspensions. The stability of graphite scrap suspensions was greatly enhanced, except for the nonionic group (Triton X-100), in which a small number of particles were able to disperse but mostly precipitate. Possibly, the functional group of Triton X-100, which consists of poly(ethylene oxide) groups, exhibits weak electrostatic repulsion. In contrast, the slurries that were stabilized by ionic dispersants (LS, SDS, CTAB, and PAA) demonstrated great stability because of their ionic segment, producing significant electrostatic repulsion. The results also showed the difference in the sedimentation level in which the suspensions containing large-molecule dispersants (LS and PAA) were higher than that of the suspensions stabilized in small-molecule dispersants (SDS and CTAB) because of differences in their structural properties. This might be a result of bridge formation between particles by a large dispersing agent, which caused aggregate formation of particles and sedimentation of aggregates.^19^ Additionally, the bubbles were observed in the suspensions stabilized by small-molecule dispersants (SDS and CTAB) due to their higher concentrations than the critical micelle concentration (CMC).

The slurry viscosity results are presented along with the stabilization test in Table 3. The highest viscosity of 450 mPas belonged to the slurry without adding additives. Without stabilization, the aggregation formation occurred in graphite scrap slurry, which caused not only unstable suspension but also high viscosity due to van der Waals forces. Adding dispersing agents can reduce viscosity and provide a flowable slurry. The viscosity of the graphite scrap slurries depended on the types of dispersing agents, which were provided as SDS < LS < CTAB < Triton X-100 < PAA with values of 60, 101, 430, 172, 291, and 448 mPas, respectively. In comparison to the other types of dispersants, the anionic group (SDS and LS) provided not only the lowest slurry viscosity but also good dispersion, which indicated high stabilization because of the strong anionic polar sulfate and sulfonate groups on their molecule. These dispersibility and viscosity results suggested that anionic dispersants (SDS and LS) were compatible with graphite scrap particles in an aqueous solvent. As a result, SDS and LS were chosen as stabilizers in ultrafine graphite scrap production by the ball milling process.

### 3.3 Ultrafine Graphite Scrap Production by the Ball Milling Process

#### 3.3.1 Effect of the Dispersant on the Ball Mill Performance of Ultrafine Graphite Scrap Production

According to its molecule size, SDS is a small anionic molecule dispersant that provides strong electrostatic repulsion but poor steric hindrance, while LS is a large anionic molecule dispersant that provides steric hindrance but weak surface activity.^20^ Therefore, the idea of mixed dispersants (LS–SDS) was introduced, and the synergistic effect of the combination was expected. In this work, single dispersants (SDS and LS) and mixed dispersants (LS–SDS) were applied in the ball milling process in order to find an optimum milling condition for the production of ultrafine graphite scrap. Particle size reduction, grinding index, and slurry appearance were used as criteria for the evaluation of milling performance. A suitable dispersant formulation should result in an accelerated reduction of particle size, a high grinding index, and a flowable slurry, all of which indicate that ultrafine graphite scrap has been well stabilized.

**Figure 3:**
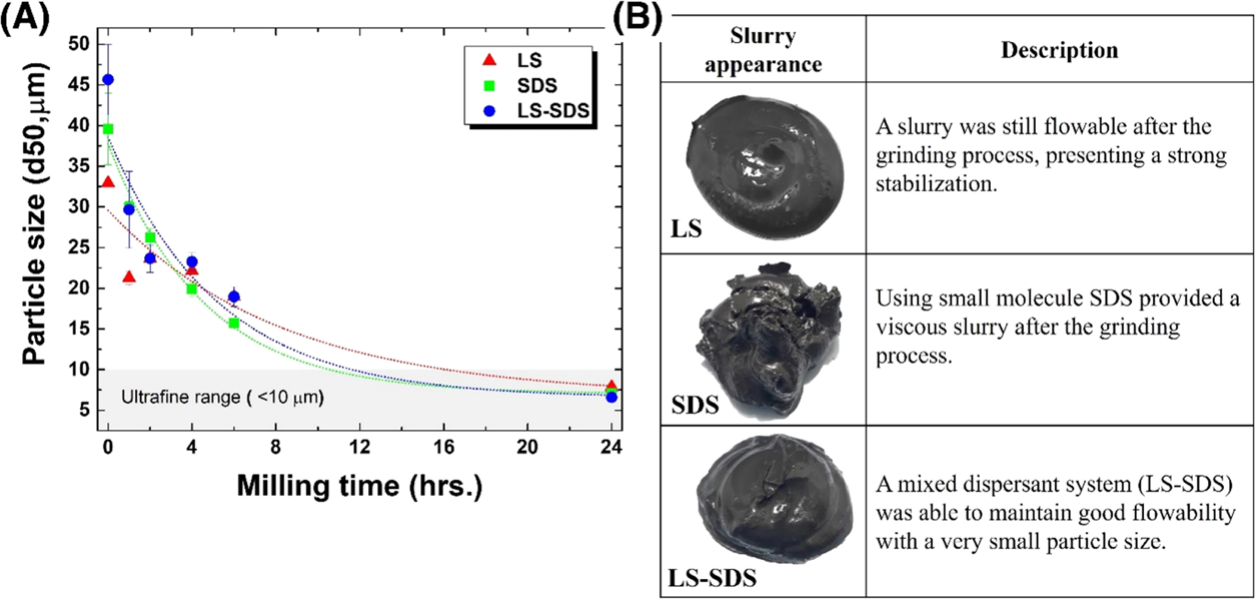
Particle size reduction along with the milling time with different dispersant conditions (A). The appearance of milled ultrafine graphite scrap slurries after the milling process for 24 h (B).

Figure 3 shows the particle size reduction and slurry appearance of single and mixed dispersant systems after the milling process. The initial particle size (*d*_50_ = 39 μm) of the raw graphite scrap was reduced to be in the ultrafine range (less than 10 μm) within 24 h. The slope of the reduction and the shorter milling time to generate ultrafine particles showed that SDS performed a greater size reduction process than LS, which was a result of the higher viscosity reduction ability of SDS, as presented in the Section 2.2. However, the produced slurry by SDS after milling was very highly viscous due to a large amount of produced ultrafine particles and insufficient stabilization from small-molecule SDS. In contrast, the milled slurry with addition of LS still provided a flowable behavior due to high enough stabilization from its large-molecule structure. The results revealed different behaviors between small-molecule (SDS) and large-molecule (LS) dispersants, in which the greater size reduction belonged to SDS and a flowable milled slurry was provided by LS. However, these two characteristics were balanced by adding the mixed dispersant formulation (LS–SDS) and the flowability of the slurry was improved with the great size reduction process similar to adding only SDS. Both size reduction and slurry appearance indicated the strong stabilization of the mixed dispersant, which was an effect of the synergistic behavior between LS and SDS. Moreover, during the same 24 h milling time, this formulation produced the smallest particles (*d*_50_ = 6.59 μm) in comparison to SDS (*d*_50_ = 7.07 μm) and LS (*d*_50_ = 7.86 μm)

**Figure 4:**
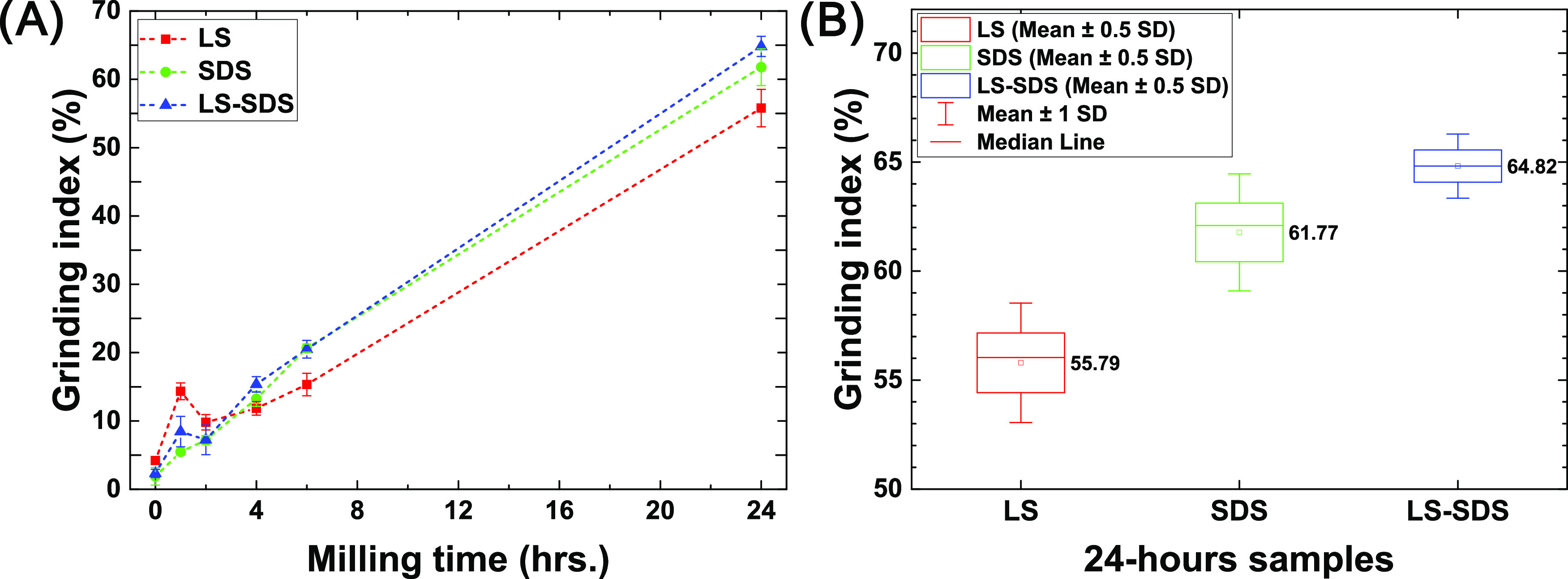
Grinding index, a quantitative milling performance of the ball milling operation, along with the milling time of the process with different dispersant formulations (A). Grinding indices of the 24 h samples (B).

The grinding index, as shown in Figure 4A, indicates the quantitative performance of the milling operation and the particle breakage event under all conditions. A high grinding index reflects a large number of fragmented particles, passing a specific size (10 μm). The results revealed an increase in the grinding index after passing the milling process, indicating an increase of ultrafine particles when milling time increased. At the beginning period, the grinding index fluctuated. The grinding index was more stable after passing for 4 h. The results showed higher grinding indices of SDS and LS–SDS than that of LS after a milling time of 4 h until the end of the process. At the same milling time of 24 h, the grinding index is in the order of LS < SDS < LS–SDS, with average values of, respectively, 56, 62, and 65% approximately, as presented in Figure 4B. The mixed LS–SDS system provided a better statistical grinding index, especially than the LS system without the overlapping of data. These grinding index results were also consistent with the particle size reduction results shown in Figure 3.

The results indicated that the mixed dispersant (LS–SDS) provided the highest milling performance in terms of size reduction, slurry appearance, and grinding index. Possibly, a synergistic effect between small (SDS) and large (LS) molecule dispersants might have occurred to stabilize particles during the milling process. SDS provided strong electrostatic repulsion, while LS offered steric hindrance, similar to the other work that used a mixed dispersant of citric acid and PAA to stabilize alumina particles through the wet grinding process.^21^ As a result, the use of a mixed dispersant formulation (LS–SDS) was suggested for the production of ultrafine graphite scrap.

#### 3.3.2 Particle Size Distribution and Morphology of Milled Ultrafine Graphite Scrap Particles

The particle size and distribution of the ultrafine graphite scrap after the ball milling process are presented in Figure 1E. The peak of size distribution shifted to the small particle size and provided a more uniform and narrow distribution in which most of the particles in the population were ultrafine particles, with sizes less than 10 μm. The low-magnification SEM image in Figure 1F displays the small pieces of graphite scrap particles, as a result of the breakage of the raw graphite material by the ball movement inside the mill chamber. In the higher-magnification SEM image, as shown in Figure 1G, the morphology of ultrafine graphite became more uniform, tiny, and flaky than the irregular raw graphite scrap, which was a result of shattering and delamination breakage mechanisms by impact and shearing force.^22^Figure 1H shows the result of the FESEM at extremely high magnification, which provides more structural information on ultrafine graphite scrap particles. The observation revealed a plate-like morphology of milled graphite scrap with an ultrafine size, a thin layer, and a smooth surface. An increase of broken bonds on the edge of the particle, as pointed by the arrow, was observed, which was a result of the shattering breakage mode. The reduction in both particle size and layer stacking of raw graphite waste caused a significant increase in the particle surface area, which was a reason for viscosity build-up in the milled slurry.

#### 3.3.3 Effect of the Dispersant on Ultrafine Graphite Scrap Characteristics

The graphite scrap was broken down into ultrafine particles after the size reduction process by using a ball mill with a varied surface treatment from a dispersing agent to control the viscosity during the milling process. Therefore, the effect of dispersants on particle characteristics such as crystalline and surface properties was investigated to identify the quality of produced ultrafine graphite scrap particles after the milling process.

The size reduction technique by ball milling utilizes the impact and shearing force of the ceramic balls to break the particles into small pieces. This particle breakage can affect the crystalline structure and induce amorphization occurring in the structure of graphite particles. The change in the crystallinity of graphite scrap by ball milling is reflected by the XRD pattern, as presented in Figure 2. The XRD results of raw graphite scrap, as received, and ultrafine graphite scrap, produced by the ball milling process, showed the same reflection pattern in all milling conditions. The width of the (002) peak was broader after milling, indicating the structural disorder occurring in the structure of ultrafine graphite scrap.^23^ This induced-amorphization by the ball milling process can be reflected by the decrease in crystalline parameters such as crystal size and graphitization degree.^24^Table 1 shows the crystalline characteristics such as *d*_002_ spacing, graphitization degree (%*G*), lateral crystalline size (*L*_a_), and stacking height (*L*_c_) of ultrafine graphite scrap with different milling conditions compared to raw graphite scrap. The results revealed an insignificant change in *d*_002_ and a slight reduction of the graphitization degree from approximately 82–81% after milling, which demonstrated the presence of a high graphitic structure of milled ultrafine graphite scrap and a small improvement of the structural disorder or amorphization during the ball milling process, possibly due to a shorter milling period and a middle milling speed with less energy input.^25^ In comparison to raw graphite scrap before milling, the lateral crystalline size (*L*_a_) decreased from 408 to 385, 378, and 365 nm in the LS, SDS, and LS–SDS conditions. The stacking height (*L*_c_), which was formerly 192 nm, was likewise reduced to 180, 172, and 155 nm. The reduction of *L*_a_ and *L*_c_ indicated the impact-induced breakdown of the in-plane structure and the shearing-induced delamination of the graphite stacking layer, corresponding to the microstructure of the graphite particles from the SEM image shown in Figure 1H, in which the particles became smaller in lateral size (along with the plane of the particle) and thinner in the layer stacking (in the vertical direction).

**Figure 5:**
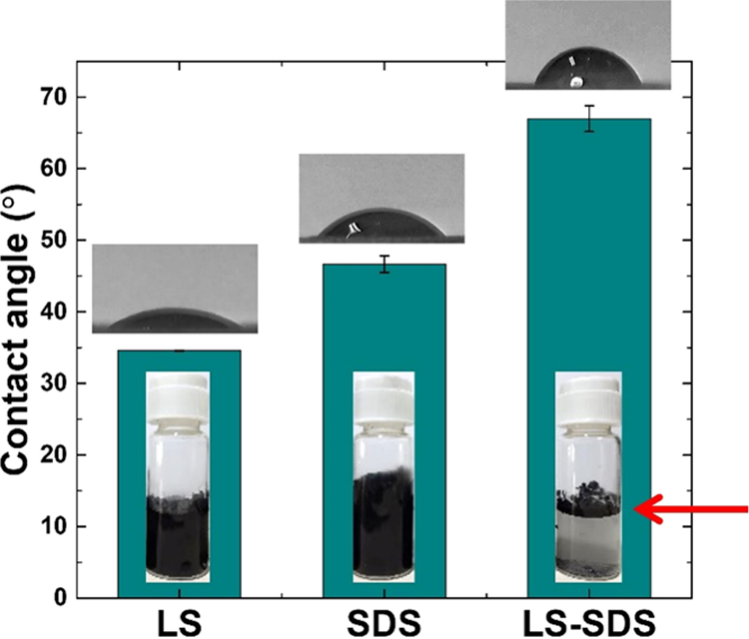
Effect of surface treatment by different dispersants on the contact angle and aqueous redispersion behaviors of milled ultrafine graphite scrap particles.

Different surface treatments using dispersant formulations in the ball milling process directly impacted the surface properties of ultrafine graphite scrap due to surface functionalization.^26^ In order to identify the surface characteristics of milled graphite scrap particles with various dispersant formulations, the water contact angles were measured along with redispersion results, as shown in Figure 5. Using LS, SDS, and LS–SDS dispersants provided angles of 34.6 ± 0.1, 46.8 ± 1.2, and 67.0 ± 1.8° respectively. The redispersion results showed that ultrafine graphite scrap particles were efficiently dispersed in a single dispersant system and floated in a mixed dispersant formulation, as pointed by the arrow. Both results demonstrated that the surface properties differed, depending on the kind of dispersant, which was a result of different dispersant structures and absorption on the particle surface.^26^ According to the contact angle results, the mixed dispersant system exhibited the highest hydrophobicity, which was consistent with the result of aqueous redispersion. This behavior might be the result of the synergistic effect and arrangement of the small- and large-molecule anionic dispersants on the graphite scrap surface.

According to the studies above, the generated ultrafine graphite scrap particles from the ball milling procedure still exhibited high crystallinity with little structural disorder. Applying the mixed dispersant (LS–SDS) yielded the highest milling performance and produced ultrafine graphite waste with the smallest crystalline size and the highest hydrophobic behavior compared to single dispersants (LS and SDS). Finally, these ultrafine graphite waste particles were used to fabricate carbon block products.

### 3.4 Fabrication of Carbon Block Products from Ultrafine Graphite Scrap

Generally, high-quality carbon and graphite blocks with high mechanical properties are often produced using small carbon fillers, which can inhibit crack formation.^6,8,27^ However, the existence of pores or voids, normally generated from the imperfect packing and volatility of the pitch binder, directly impacts the decrease of mechanical properties. These pores can be filled by using impregnation, which upgrades the product to higher quality. According to the report, the impregnation technique increased product density by 2–2.5% and reduced porosity by 15–24%.^28^ Herein, the carbon blocks were fabricated from ultrafine graphite scrap through the carbonization process and upgraded to a higher quality by the impregnation process. The microstructure of the carbon block before and after impregnation was presented. The product properties, such as density, hardness, flexural strength, and electrical resistivity, were measured. Additionally, the effect of dispersant formulations on the product properties confirms the potentiality of using dispersant formulations in the actual process.

**Figure 6:**
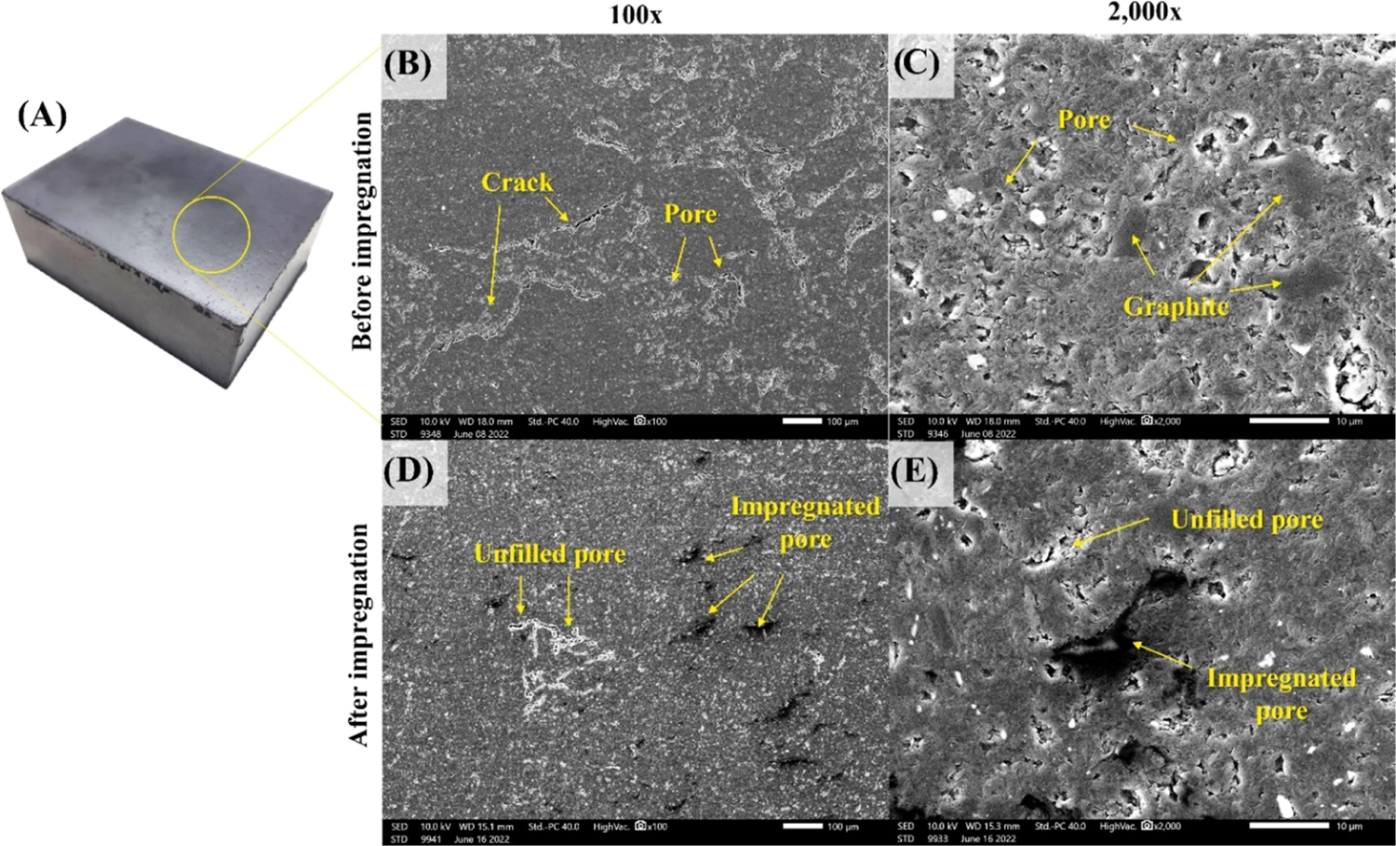
Appearance of carbon block products after the manufacturing process (A). Microstructure of the carbonized carbon block after polishing (B, C) and the impregnated carbon block (D, E) at different magnifications.

Carbon block products from milled ultrafine graphite waste after the manufacturing processes were achieved as presented in Figure 6A. The microstructures of the carbonized carbon blocks were characterized by the SEM image at different magnifications (Figure 6B,C), in which the appearance of cracks, pores, and graphite scrap particles was pointed by the arrow. The results demonstrated the broad pore size distribution and the formation of cracks by the connection of these pores. The existence of these pores and cracks directly reduces the mechanical properties.^2^ However, the occurrence of pores was normally observed, which was caused by the imperfect packing of carbon blocks and the volatility of organic molecules from the coal tar pitch binder at high temperatures.^29^ The observation also revealed the embedding of ultrafine graphite scrap particles in the carbon block, as pointed by the arrow in Figure 6C. The particle size of the embedded ultrafine graphite scrap was less than 10 μm, which is consistent with the particle size results of milled ultrafine graphite particles, with a *d*_50_ of roughly 6.22 μm. These results confirmed the achievement of the carbon block products, fabricated from the ultrafine graphite scrap.

The SEM images in Figure 6D,E illustrate the microstructure of carbon blocks after impregnation at different magnifications. The pores of the samples were filled with an impregnating chemical, as pointed by the arrow. The results revealed the white area of unfilled pores, which possibly are closed pores, and the black area of filled pores; the edges of the pores were hardly visible. The impregnation had filled not only the large pores but also the microscopic pores, as observed in Figure 6E. As a result, the impregnation process filled the pores of the carbon block samples, causing denser carbon block materials.

**Figure 7:**
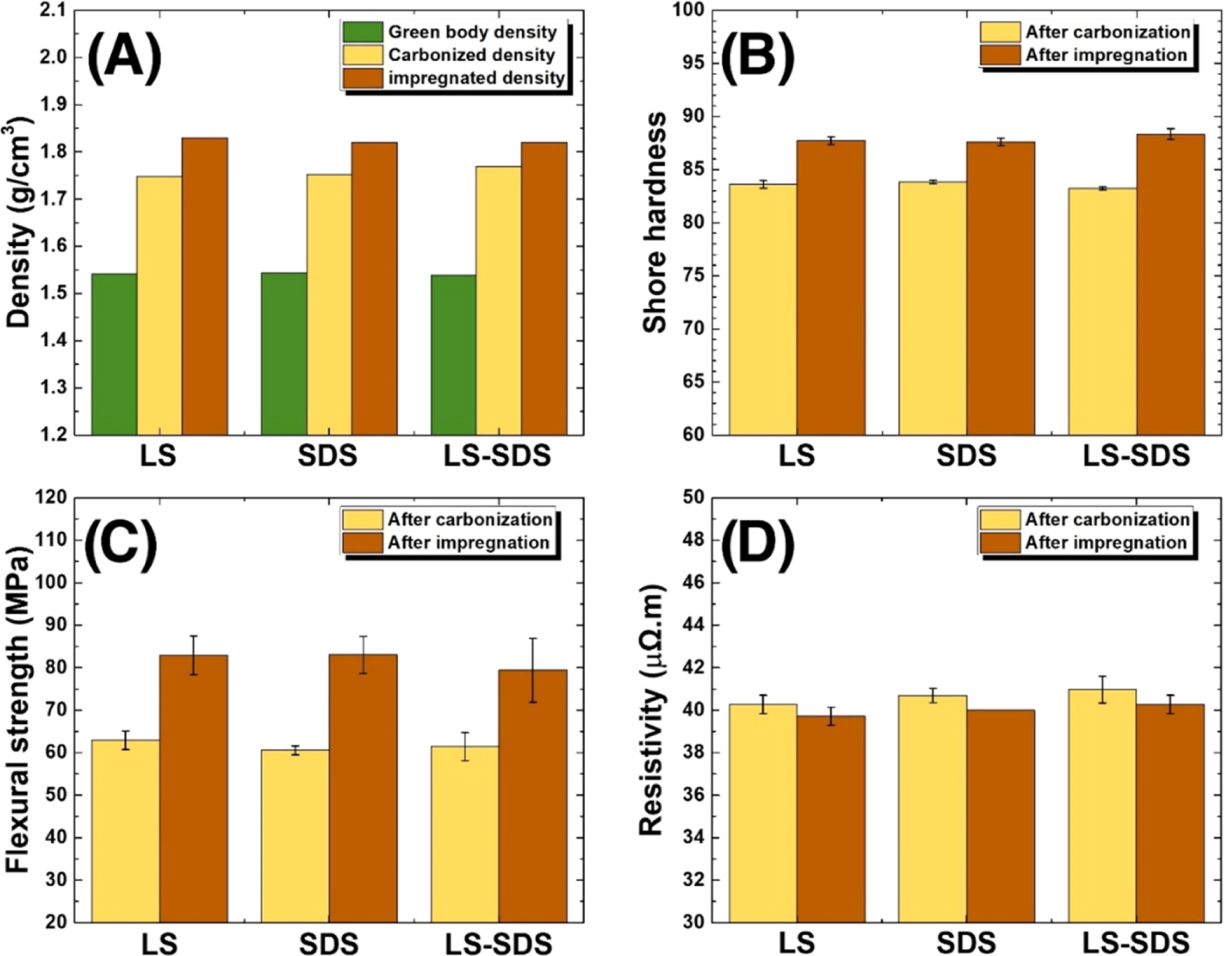
Product properties of the carbonized carbon block and the impregnated carbon block, fabricated from milled ultrafine graphite scrap with different dispersant conditions: density (A), shore hardness (B), flexural strength (C), and electrical resistivity (D).

The product properties in terms of density, hardness, flexural strength, and electrical resistivity of carbon blocks before and after the impregnation process are presented in Figure 7 along with dispersant conditions used in the ball milling process to produce ultrafine graphite scrap. The densities of the green body block and the carbonized carbon block were about 1.54–1.55 and 1.75–1.77 g/cm^3^, respectively, as represented in Figure 7A. The increase of density after carbonization at 950 °C was a result of densification, which was caused by a slight weight loss from the volatile matter of the pitch binder and the volumetric shrinkage of the green body block.^2,29^ According to the results of product properties in Figure 7B–D, the flexural strength, shore hardness, and electrical resistivity of carbonized carbon blocks were evaluated to ∼61–63 MPa, 83–84, and 40.3–41 μΩ m, respectively.

The product properties were also developed by using an impregnation procedure. The results revealed an increase in density by around 5% to 1.82–1.83 g/cm^3^, corresponding to the microscopic structure from SEM images shown in Figure 6D,E. Hardness increased from 83.2–83.8 to 87.6–88.3, an ∼6% increase, while flexural strength increased from 60.6–62.9 to 79.4–82.9 MPa, a 40% improvement. These better mechanical properties were a result of a denser structure by filling the pores with impregnation.^2,29^ Additionally, electrical resistivity was slightly reduced by around 1.6% from 40.3–41.0 to 39.7–40.3 μΩ m, which was a result of a nonconductive impregnating agent.

Following the surface treatment results, the different dispersant formulations in ultrafine graphite scrap production by the ball milling process presented different surface properties, especially the mixed dispersant system. However, the result of product properties remained within a similar range, even though the hydrophobicity of the ultrafine graphite waste was varied by dispersion agents. This demonstrated the negligible impact of surface treatments with LS, SDS, and LS–SDS on the product properties, which might be a result of using a low dispersant concentration and degradation of dispersants at high temperatures. As a result, the mixed dispersing agent (LS–SDS) with the most effective mill performance was available to produce ultrafine graphite waste for carbon block products.

The usage of ultrafine graphite scrap as a carbon filler has the potential for circular economic production of carbon blocks. The milling process with high milling performance by using mixed dispersant formulations (LS–SDS) was available to fabricate ultrafine graphite scrap without the effect on the properties of the carbon block products. The properties of fabricated carbon block products were also comparable to those of typical carbon products, having a density of 1.8 g/cm^3^, a hardness range of 90–95, and a flexural strength range of 76–79 MPa.^30^ Therefore, the carbon blocks made from graphite waste could be competitive with carbon products from typical production.

## 4 Conclusions

Carbon block products made from ultrafine graphite scrap were achieved. The ball milling process for ultrafine graphite scrap production was developed using dispersing agents. In comparison to a single anionic dispersant (LS and SDS), the mixed dispersant (LS–SDS) provided the highest milling performance, as monitored by rapid size reduction with the smallest particle size, with a *d*_50_ of 6.22 μm, at a milling time of 24 h, the highest grinding index at 65%, and a flowable milled slurry, which was a result of the synergistic interaction of small- and large-molecule dispersants. The ultrafine graphite scrap with different dispersants could be used to fabricate carbon block products with negligible impact on the properties, indicating the potential use of mixed dispersant formulations (LS–SDS) in actual carbon block production. The fabricated carbon block provided estimated density, hardness, flexural strength, and electrical resistivity values of 1.76 g/cm^3^, 83, 62 MPa, and 40 μΩ m, respectively, and the properties were improved by the impregnation process to be 1.83, 88, 81 MPa, and 39 μΩ m, respectively, which could be comparable to the properties of typical carbon products.
